# Clinical Outcomes of Peripheral Magnetic Stimulation in Patients with Functional Defecation Disorders

**DOI:** 10.3390/healthcare14203377

**Published:** 2026-10-10

**Authors:** Wenxuan Ma, Chengjing Feng, Manman Fan, Wenqing Gu, Caiyun Peng, Min Ni

**Affiliations:** 1 Graduate School, Nanjing University of Chinese Medicine, Nanjing 210000, China; wenxuam@njucm.edu.cn (W.M.); 15256941433@163.com (C.F.); 18112557548@163.com (M.F.); guwenqing@njucm.edu.cn (W.G.); pengcaiyun@njucm.edu.cn (C.P.); 2 Nanjing Hospital of Chinese Medicine Affiliated to Nanjing University of Chinese Medicine, Nanjing 210000, China

**Keywords:** functional defecation disorders, peripheral magnetic stimulation, constipation, high-resolution anorectal manometry, anorectal sensorimotor function

## Abstract

**Aim:** This article aims to evaluate the therapeutic efficacy of peripheral magnetic stimulation (PMS) and its effects on anorectal sensorimotor function in patients with functional defecation disorders. **Methods:** This uncontrolled retrospective study reviewed the clinical data of patients diagnosed with FDD according to the Rome IV criteria who received PMS. Constipation symptoms and quality of life were assessed using the constipation scoring system (CSS) and the patient assessment of constipation quality of life (PAC-QOL) questionnaire before and after treatment. Secondary outcomes included anorectal motor and sensory function assessed by high-resolution anorectal manometry (HRAM), including anorectal pressure parameters, rectal sensory thresholds, and rectal anal inhibitory reflex (RAIR). **Results:** Following treatment, significant improvements were observed in constipation symptoms and quality of life. CSS and PAC-QOL scores were significantly reduced compared with baseline (both *p* < 0.05). Among the individual CSS items, significant improvements were observed in defecation straining and time spent in the lavatory per attempt (both *p* < 0.05). HRAM demonstrated significant reductions in anal resting pressure, anal residual pressure during simulated defecation, and anorectal pressure gradient, accompanied by a significant increase in anal relaxation rate (all *p* < 0.05). Rectal sensory function was also improved, as evidenced by a significant reduction in the maximum tolerable rectal volume (*p* = 0.021). Although no significant changes were observed in RAIR parameters, a trend toward improved internal anal sphincter relaxation was noted. PMS was generally well tolerated, and no serious treatment-related adverse events were reported. **Conclusions:** In this uncontrolled retrospective study, patients with FDD showed significant improvements in constipation symptoms, quality of life, anorectal motility, and rectal sensory function following PMS treatment. The observed changes in anorectal motility and rectal sensation suggest that PMS may improve defecatory dysfunction through modulation of anorectal sensorimotor function. Prospective controlled studies are warranted to further confirm these findings.

## 1. Introduction

Chronic constipation (CC) is a common condition affecting the gastrointestinal system. The global pooled prevalence of CC was estimated at 14% based on a large meta-analysis [1]. The Rome IV criteria categorized patients with primary CC (not attributed to another disease) as having functional constipation (FC), constipation-predominant irritable bowel syndrome (IBS-C), or functional defecation disorders (FDD) [2]. FDD are a kind of acquired dyssynergic defecation, with the following pathophysiological characteristics: paradoxical contractions of the anorectum or inadequate relaxation of the pelvic floor muscles during attempted defecation and/or inadequate propulsive forces during attempted defecation and/or altered rectal sensation [3,4]. The prevalence of FDD in the general population is unknown, although it can be detected in 27–59% of patients with CC [5], indicating that functional defecation disorders are a common clinical gastrointestinal disorder.

Based on the pathophysiology of FDD, biofeedback is the mainstay of treatment for FDD, with the function of coordinating abdominal and pelvic floor motion during evacuation [2]. However, its widespread clinical application is limited by the requirement for specialized equipment, trained personnel, prolonged treatment courses, and high patient compliance. Initial management of chronic constipation generally includes lifestyle and dietary modifications, fiber supplementation, and laxative therapy [1,6]. Patients subsequently diagnosed with FDD often receive these conservative measures before or in conjunction with specialized anorectal therapies in routine clinical practice, particularly in settings where biofeedback therapy is not readily available. Nevertheless, pharmacological therapies primarily alleviate constipation symptoms and do not directly address the underlying anorectal dysfunction, resulting in variable therapeutic efficacy.

Peripheral magnetic stimulation (PMS) is a safe and non-invasive technique according to Faraday’s law of electromagnetic induction. It utilizes a magnetic coil to generate a pulsed electromagnetic field that penetrates tissues to have the effects of modulating muscle strength, improving blood circulation, and inducing nerve impulses, with a wide and deep range of stimulation [7,8]. This modality received approval from the United States Food and Drug Administration in 1998 for the management of urinary incontinence, and it can be applied to other pelvic floor dysfunction disorders [9]. In 1998, Shafik found that magnetic stimulation of the sacral nerve can activate the rectal muscles, enhance intestinal motility, and facilitate defecation in patients with chronic constipation [10]. The studies conducted by Chiu et al. and Huang et al. proved that PMS improves constipation in patients with Parkinson’s disease, potentially by reducing colonic transit time and increasing the anorectal angle and pelvic floor descent during evacuation [11,12]. Yun et al. reported that transabdominal functional magnetic stimulation can ameliorate constipation symptoms in individuals with brain injury [13]. However, none of the previous studies have explored the role of PMS in the management of FDD.

The aim of this study was to explore the efficacy of PMS on constipation and anorectal motor and sensory functions in patients with FDD.

## 2. Materials and Methods

### 2.1. Study Design

This uncontrolled retrospective cohort study was conducted at the Department of Colorectal and Anal Surgery, Nanjing Hospital of Traditional Chinese Medicine. Clinical data of patients diagnosed with FDD according to the Rome IV criteria between January 2024 and December 2025 were retrospectively reviewed. The diagnosis of FDD was established based on clinical manifestations, balloon expulsion test (BET), and high-resolution anorectal manometry (HRAM). The diagnostic criteria and anorectal physiological assessment were based on the consensus recommendations of the International Anorectal Physiology Working Group (IAPWG) [14].

Patients who completed the PMS treatment course and had complete clinical and anorectal physiological data before and after treatment were included in the analysis.

### 2.2. Inclusion and Exclusion Criteria

The inclusion criteria were as follows:

(1) Diagnosed with FDD in accordance with the Rome IV criteria [15]:

Criteria was fulfilled for the last 3 months with symptom onset at least 6 months prior to diagnosis.

① Met diagnostic criteria for functional constipation and/or irritable bowel syndrome with constipation (IBS-C):

Functional constipation: Patients were required to have at least two of the following symptoms in more than 25% of defecations: (a) straining; (b) lumpy or hard stools (Bristol Stool Form Scale types 1–2); (c) sensation of incomplete evacuation; (d) sensation of anorectal obstruction or blockage; (e) manual maneuvers to facilitate defecation; or (f) fewer than three spontaneous bowel movements per week. Loose stools were rarely present without the use of laxatives, and the patient did not fulfill the diagnostic criteria for IBS.

IBS-C: Patients were required to have recurrent abdominal pain, occurring on average at least 1 day per week during the preceding 3 months, associated with at least two of the following: (a) related to defecation; (b) associated with a change in the frequency of stool; and (c) associated with a change in the form (appearance) of stool. In addition, IBS-C was characterized by predominantly hard or lumpy stools (Bristol Stool Form Scale types 1–2) and rarely loose or watery stools (types 6–7), according to the Rome IV criteria.

All information regarding these symptoms was obtained from the standardized medical history and clinical interview conducted at the initial patient consultation.

② During repeated attempts to defecate, there must be features of impaired evacuation, as demonstrated by 2 of the following 3 tests:

a. Abnormal balloon expulsion test
b. Abnormal anorectal evacuation pattern with manometry
c. Impaired rectal evacuation by imaging.

(2) 18–85 years of age.
(3) Completed the PMS treatment course
(4) Availability of complete symptom, quality of life, psychological, and anorectal physiological data before and after treatment.

The exclusion criteria were as follows:

(1) Constipation secondary to identifiable organic or structural anorectal diseases that could affect defecatory function.
(2) Major neurological disorders that could affect anorectal sensorimotor function, including Parkinson’s disease, cerebrovascular disease, spinal cord lesions, multiple sclerosis, or clinically significant diabetic neuropathy.
(3) Constipation secondary to identifiable metabolic or systemic diseases.
(4) Inability to complete the prescribed PMS treatment course.
(5) Incomplete clinical, quality-of-life, psychological, or anorectal physiological data required for the analysis.

### 2.3. Intervention

**Figure 1:**
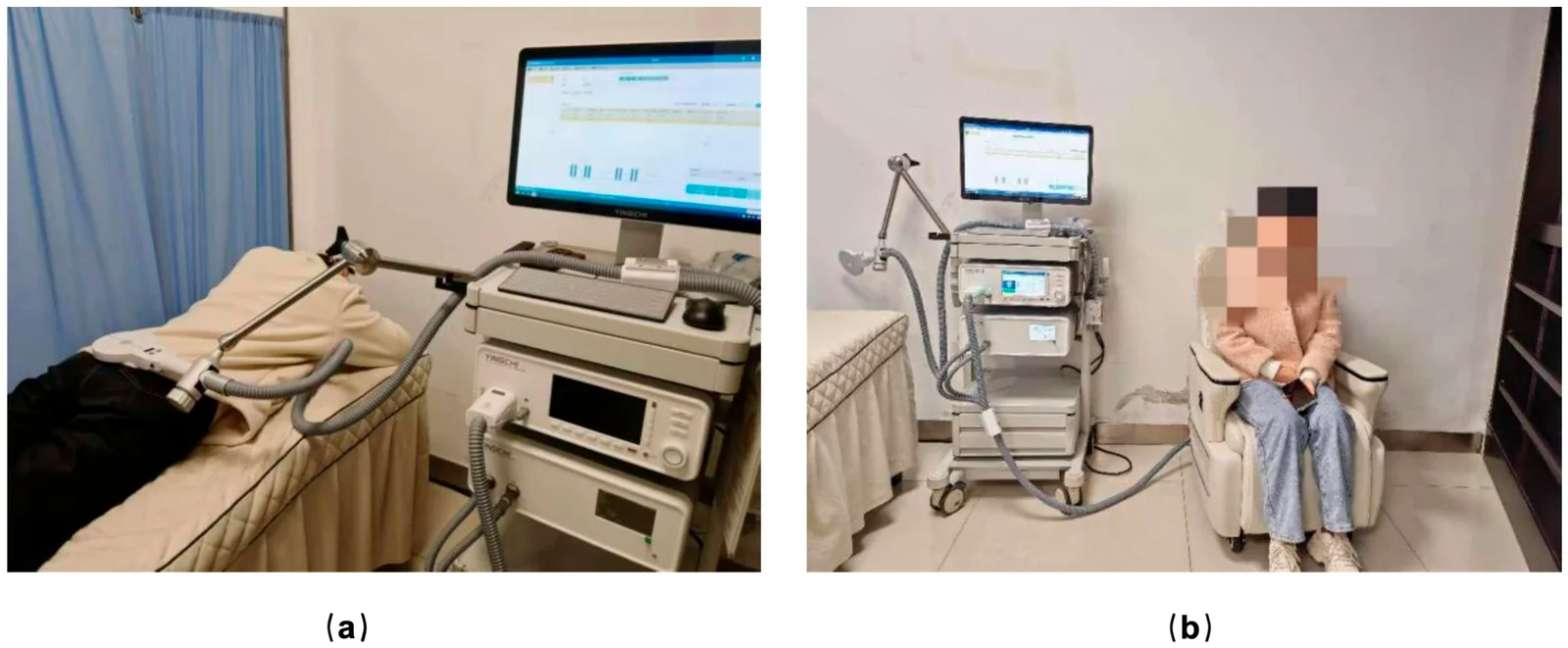
Procedure of PMS. (**a**) Lumbosacral magnetic stimulation; (**b**) Pelvic floor magnetic stimulation in a patient.

Patients received PMS treatment with a magnetic stimulation device (Model: M-100 Ultimate, YINGCHI, Shenzhen, China). PMS was delivered at 2 sites (anus and lumbosacral S2–S4 region), with each site lasting 40 min. Patients were seated in a specialized treatment chair with an embedded stimulation coil and were instructed to position themselves to align their anus with the point of maximal stimulation, as determined by their sensation. The stimulation frequency was set at 1 Hz based on previous evidence from Rao et al., which showed that 1 Hz stimulation was more effective than higher-frequency stimulation in improving anorectal physiological parameters [16,17,18]. Figure 1 illustrates the treatment setup. The treatment was administered once a day, five times a week for a total of two weeks.

### 2.4. Outcomes and Evaluation

#### 2.4.1. Constipation Symptoms and Quality of Life Score

Participants completed the constipation scoring system (CSS) and patient assessment of constipation quality of life (PAC-QOL) before and after the treatment [19,20]. CSS was developed in 1996 by Feran Agachan. It is used to evaluate constipation severity, including 8 items: defecation frequency, abdominal symptoms, defecation time, unsuccessful attempts, and history of constipation. It has advantages in evaluating patients’ condition, guiding treatment, and assessing the effectiveness of interventions.

The total CSS score was calculated by summing all eight component items, ranging from 0 to 30. Quality of life was evaluated using the PAC-QOL questionnaire, which comprises 28 items, including worriedness and concerns, physical discomfort, psychosocial discomfort, and satisfaction in daily life. Higher scores in the two questionnaires indicated greater constipation symptoms and worse quality of life.

#### 2.4.2. Psychological Assessment

Hamilton anxiety scale (HAMA, 14-item version) and Hamilton depression scale (HAMD, 24-item version) were applied to assess mental status [21,22].

HAMA scores were interpreted as follows: values of 7–13 indicate possible or mild anxiety, 14–20 correspond to definite or moderate anxiety, and scores ≥ 21 reflect severe anxiety. For HAMD, scores of 8–16 denote mild depression, 17–23 represent moderate depression, and values ≥ 24 indicate severe depression.

#### 2.4.3. High-Resolution Anorectal Manometry

High-resolution anorectal manometry (HRAM) was conducted in both groups before and after the intervention using an eight-channel water-perfused high-resolution manometry system (Model: GAP-24A; Mida Medical Instruments Co., Ltd., Ningbo, China).

Patients were required to defecate before the test. If residual stool was found in the rectum upon digital rectal examination, two glycerin enemas were administered. HRAM was performed 30 min after the enemas were given.

Patients were positioned in the left lateral decubitus position with their hips and knees flexed at a 90-degree angle. A digital rectal examination was performed prior to manometry. After calibrating and zeroing the manometry system, a lubricated catheter with an integrated balloon was gently inserted through the anus into the rectum. Then the balloon was inflated with 50 mL of air, and the patient was asked to expel the balloon. If the balloon was not expelled within 1 min, it was considered a failure, indicating an abnormal balloon expulsion test. Then, the catheter was repositioned appropriately and HRAM was performed to derive the following parameters: resting pressure, squeeze pressure, defecation pressure, sensation threshold, and rectoanal inhibitory reflex (RAIR) [14]. RAIR was defined as a reduction exceeding 25% of the anal resting pressure in response to rapid rectal balloon inflation occurring when the balloon reached a specific threshold volume or any volume above it [23].

A review of the medical records showed that the CSS, PAC-QOL, and HRAM were reassessed within 3 days after completion of the final treatment session, with slight variations in timing according to patients’ schedules and clinical arrangements.

### 2.5. Statistical Analyses

All statistical analyses were performed using SPSS (version 25.0, IBM Corp., Armonk, NY, USA). The normality of continuous variables was evaluated with the Shapiro–Wilk test. Data conforming to a normal distribution are expressed as mean ± standard deviation (mean ± SD). Variables that did not meet normality assumptions are presented as median and interquartile range [median (QL, QU)]. For such data, the Wilcoxon signed-rank test was used. A two-sided *p*-value < 0.05 was considered statistically significant.

## 3. Results

### 3.1. Baseline Characteristics

A total of 21 patients were included in the study. Among them, 12 (57.1%) were male and nine (42.9%) were female. The mean age of the patients was 57.71 ± 15.46 years, and the mean disease duration was 5.86 ± 3.75 years. Detailed demographic and clinical characteristics are summarized in Table 1.

### 3.2. Effects of PMS on Constipation

**Figure 2:**
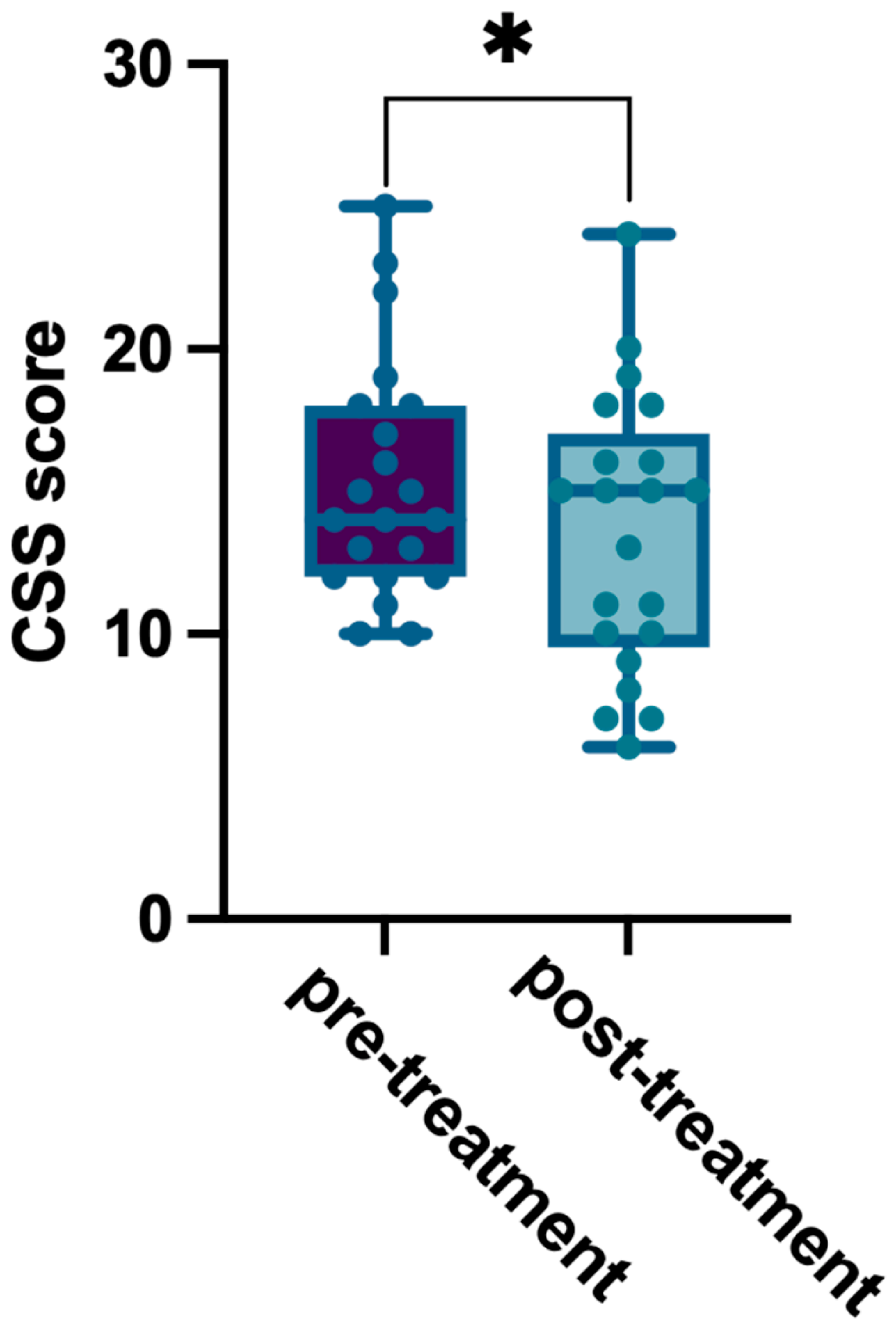
Changes in total CSS score before and PMS treatment. CSS score was significantly decreased after treatment compared with baseline. *—*p* < 0.05.

Following the two-week intervention, the total constipation scoring system (CSS) score was significantly reduced compared with baseline (*p* < 0.05; see Table 2 and Figure 2). Significant improvements were observed in defecation straining (*p* = 0.004) and time spent in the lavatory per attempt (*p* = 0.038), which contributed substantially to the overall reduction in CSS scores.

However, no significant changes were noted for other symptoms, including the sense of incomplete evacuation, abdominal pain, the need for defecation assistance, or the frequency of unsuccessful attempts (all *p* > 0.05).

Since all patients had a defecation frequency score of 0 both before and after treatment, and the disease duration remained unchanged throughout, these two items were not included in the comparison of the detailed scoring components.

### 3.3. Effects of PMS on Quality of Life and Anxiety/Depression

**Figure 3:**
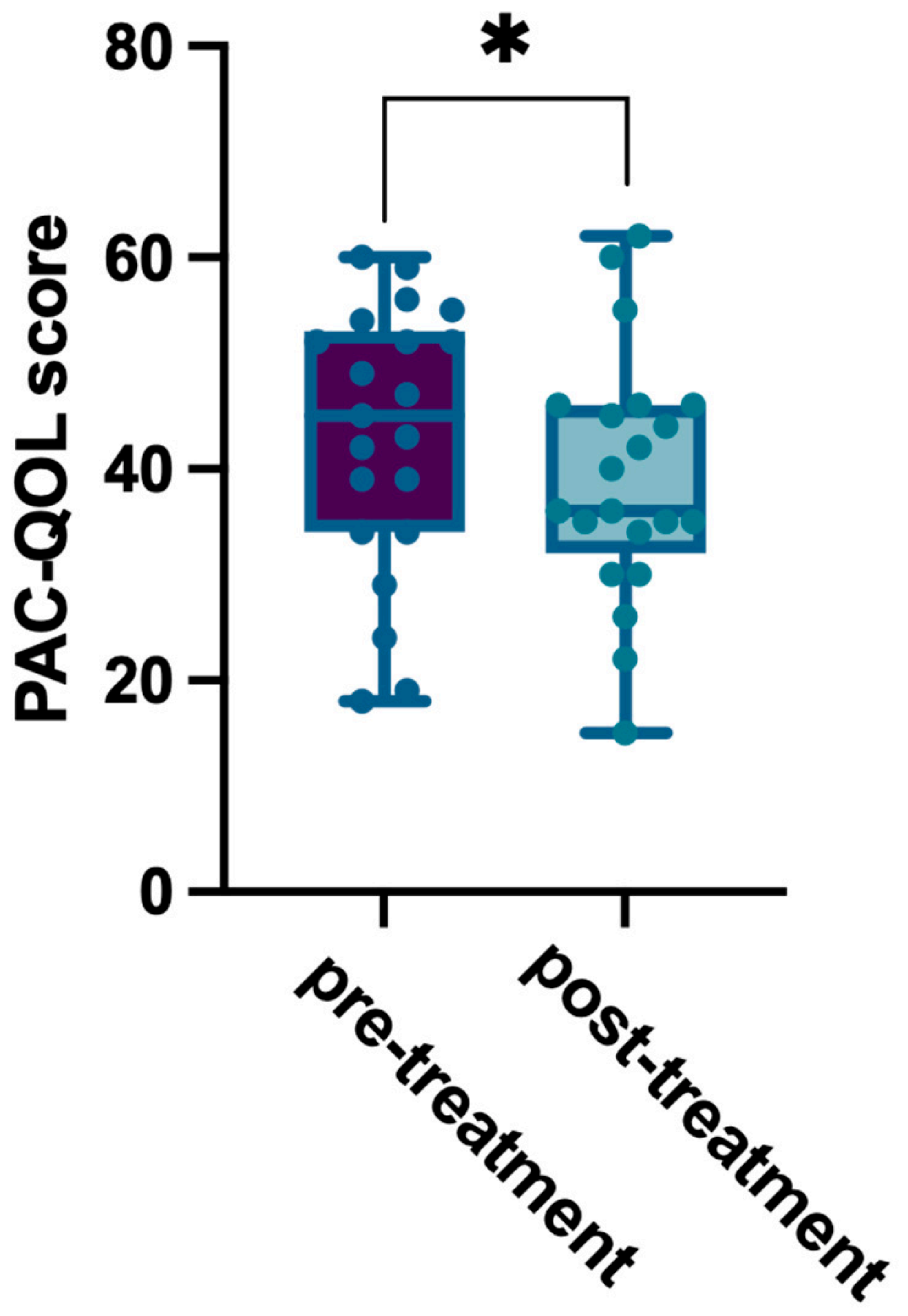
Changes in PAC-QOL score before and after PMS treatment. PAC-QOL score significantly decreased after treatment compared with baseline. *—*p* < 0.05.

The score of PAC-QOL was decreased after PMS (*p* < 0.05) (Figure 3). No significant changes were noted in the HAMA and HAMD compared with baseline.

### 3.4. Effects of PMS on Anorectal Motility

Following treatment, a significant decrease was observed in anal resting pressure, anal residual pressure during simulated defecation, and anorectal gradient, while the anal relaxation rate was markedly increased in comparison with baseline (all *p* < 0.05; see Table 3). Detailed results are presented in Table 3. No significant changes were observed in maximum anal squeeze pressure or rectal defecation pressure following treatment (both *p* > 0.05).

### 3.5. Effects of PMS on Rectal Sensation and RAIR

Following treatment, anorectal sensory thresholds were reassessed using HRAM (Table 4). A significant reduction in the maximum tolerable rectal volume was observed compared with baseline (*p* = 0.021), whereas no significant changes were found in the first sensation volume, desire-to-defecate volume, or urge-to-defecate volume (all *p* > 0.05).

No statistically significant changes were observed in any RAIR parameters following treatment (all *p* > 0.05). However, a trend toward improved RAIR function was noted, characterized by a decrease in RAIR residual pressure (40.29 ± 22.08 mmHg vs. 32.33 ± 16.48 mmHg) and an increase in the RAIR relaxation rate (0.49 ± 0.17 vs. 0.54 ± 0.17).

### 3.6. Safety and Tolerability

Treatment was generally well tolerated. Five patients experienced mild and transient adverse events during the initial treatment sessions, including transient discomfort (*n* = 2) and stimulation site irritation (*n* = 3), all of which resolved spontaneously within 5–10 min without intervention. No treatment discontinuation or serious adverse events were reported.

## 4. Discussion

In the clinical study, PMS significantly improved constipation symptoms and quality of life in patients with FDD, as evidenced by reductions in CSS scores and improvements in PAC-QOL scores. HRAM demonstrated that PMS significantly enhanced anorectal motility and sensation, including anal residual pressure, anal relaxation rate, and maximum tolerable volume. These findings suggest that PMS may represent a safe and promising therapeutic approach for improving anorectal dysfunction in patients with FDD.

PMS was applied to the anus and sacrococcygeal regions in the study. The pulsed electromagnetic fields produced by the device can penetrate deeply into the pelvic floor musculature and induce depolarization of motor neurons, eliciting strong and sustained muscle contractions [9,24]. Stimulation at the anal region was designed to target the downstream uncoordinated pelvic floor muscles, particularly the external anal sphincter and puborectalis, which are key effectors in dyssynergic defecation [25]. The principal somatic and autonomic innervation of the pelvic floor musculature, rectum, and anus arises from the S2–S4 sacral nerve roots, and targeting these segments represents an effective strategy for regulating pelvic organ function and modulating pelvic floor activity [24,26]. The sacrococcygeal stimulation targets the S2–S4 roots, possibly mediating sphincter muscle function and regulating rectal sensitivity and contractility, and anal motility [27]. Taken together, the combination of the two sites provided a comprehensive treatment that targets the pathophysiology of FDD.

A frequency of 1 Hz was utilized in the study. It is based on several pieces of clinical evidence indicating that low-frequency magnetic stimulation is effective in modulating anorectal neuromuscular function. The study conducted by Algladi et al. demonstrated that 1 Hz lumbosacral magnetic stimulation enhanced spinal-level neuroplasticity, leading to changes in anal sphincter excitability and improving anorectal sensation [17]. Leung et al. reported that magnetic stimulation with 0.5 Hz promotes the recovery of peripheral nerve injuries and normalizes sensation [18]. Rao et al. conducted a randomized frequency-response trial comparing 1 Hz, 5 Hz, and 15 Hz translumbosacral neuromodulation and found that 1 Hz stimulation produced superior improvements in anorectal neuropathy, anal squeeze pressure, and rectal capacity, along with a significantly higher responder rate compared with higher frequencies [16]. Overall, these findings support 1 Hz as an optimal stimulation frequency.

In the study, the improvement of constipation symptoms and quality of life was measured by CSS and PAC-QOL. The scores of CSS and PAC-QOL showed significant improvement. Symptom improvement was predominantly characterized by reduced defecation straining and shorter time spent during defecation, which are clinically relevant manifestations of impaired anorectal coordination in patients with FDD [28]. Our findings are consistent with previous studies investigating PMS for constipation. Several studies applied the Knowles–Eccersleye–Scott Symptom (KESS) Questionnaire, which is a validated symptom scoring system showing strong positive correlation with CSS, to assess constipation severity [29]. Chiu et al. and Wang et al. conducted longitudinal studies and revealed a reduction in KESS score after magnetic stimulation, supporting the potential beneficial effects of magnetic stimulation on constipation symptoms [11,30]. Similarly, Huang et al. also reported that 4-week magnetic stimulation at the sacrum led to significant improvements in KESS scores and PAC-QOL from baseline in the treatment group [12]. These findings support the beneficial effects of magnetic stimulation on symptom burden and quality of life and are in agreement with the results of the present study. Although the CSS and PAC-QOL significantly decreased after treatment, the absolute reduction was relatively modest. Therefore, the statistical significance observed in this study should not be interpreted as definitive evidence of a clinically meaningful improvement. Currently, there is no universally accepted and minimal clinically important difference for the CSS and PAC-QOL that can be directly applied to patients with functional defecation disorders. Therefore, larger prospective studies with longer follow-up are warranted to further determine the clinical significance and durability of the observed changes.

The dysfunction of anorectal motility is an important phenomenon and pathophysiological factor for FDD [31]. The present study demonstrated that PMS alleviated anorectal motor parameters, including reductions in anal resting pressure and anal residual pressure during simulated defecation, as well as increases in anal relaxation rate and anorectal pressure gradient. These parameters are important indicators of anorectal coordination and evacuation efficiency. These findings suggest that PMS may exert regulatory effects on anorectal motor function rather than merely alleviating subjective constipation symptoms. Consistent with our observations, Wang et al. and Chiu et al. reported an increase in the anorectal angle during evacuation after PMS treatment, a change that is closely related to improvement in the anorectal pressure gradient and facilitates effective defecation [11,30,32]. Although anorectal angle and anorectal pressure gradient represent different physiological parameters, both provide information regarding the coordination and effectiveness of anorectal evacuation. The improvement in anorectal pressure dynamics observed in the present study therefore provides complementary physiological evidence supporting the potential effects of magnetic stimulation on defecatory function. However, not all previous findings are consistent with those of the present study. Fawaz SI et al. combined lumbosacral magnetic stimulation with biofeedback as a treatment for functional constipation [33]. Magnetic stimulation was associated with increased anal resting pressure and reduced maximum squeeze pressure, which are not entirely consistent with those of the present study. These discrepancies may be explained by differences in stimulation parameters and patient characteristics. While Fawaz SI et al. enrolled patients with all subtypes of functional constipation, our study focused specifically on patients with FDD. Moreover, the use of low-frequency PMS (1 Hz) in the present study, as opposed to high-frequency stimulation (20 Hz) in their study, results in different neuromuscular responses. Taken together, these findings indicate that PMS improves defecatory function in patients with FDD by modulating anorectal motility and coordination, particularly through enhancing anorectal pressure dynamics.

Rectal sensation is also critical to normal defecation by allowing individuals to perceive rectal filling and initiate appropriate defecatory responses. Adequate rectal filling and intact sensory function activate rectal afferent pathways and generate the perception of rectal fullness [34]. Impaired rectal sensation is associated with more serious constipation symptoms [35]. In the study, a significant enhancement of rectal sensory perception after PMS treatment was shown. This was evidenced by a post-treatment reduction in the maximum tolerable volume (*p* < 0.05). Rao et al. reported that, in patients with fecal incontinence, PMS treatment led to significant increases in rectal sensory thresholds for constant sensation, urge to defecate, and maximal tolerable volume compared with baseline, which differs from our results [16]. The difference may be blamed on the disease-specific baseline. Taken together with our findings, it is indicated that PMS may exert a modulatory effect on rectal sensation. However, direct evidence supporting the effects of PMS on rectal sensation in patients with constipation is limited. Our findings provide preliminary clinical evidence supporting its potential role in modulating anorectal sensory pathways. Impaired nerve conduction and localized rectal lesions diminish the perception of mechanical distention, leading to weakened desire to defecate and urge to defecate [36,37]. PMS may directly stimulate sensory nerve endings in the rectal wall and sacral nerve roots, which in turn modulates somatosensory pathways and helps restore the suppressed defecation reflex to improve rectal sensitivity [38,39].

From a clinical and practical perspective, biofeedback therapy remains the established first-line treatment for FDD because it directly targets impaired pelvic floor coordination through individualized sensorimotor training. PMS, in contrast, is a non-invasive neuromodulatory approach that does not require active pelvic floor coordination during stimulation. Biofeedback requires appropriate anorectal physiological assessment and trained therapists, whereas PMS requires dedicated magnetic stimulation equipment and is administered by trained healthcare professionals. The two approaches may therefore differ in equipment requirements, personnel involvement, treatment duration, patient burden, and accessibility across healthcare settings. However, the present study did not prospectively collect health-economic data, including direct treatment costs, equipment and personnel costs, adherence-related costs, or long-term healthcare utilization. Consequently, a formal cost-effectiveness comparison between PMS and biofeedback cannot be made based on the present data. Given the current evidence, PMS should not be regarded as a replacement for biofeedback therapy but may represent a potential adjunctive or alternative option for selected patients, particularly when biofeedback is unavailable, poorly tolerated, or difficult to implement. Future randomized controlled trials should incorporate standardized health-economic assessments, treatment adherence, patient-reported treatment burden, and long-term outcomes to better define the clinical and economic position of PMS within the treatment algorithm for FDD.

The present study has several strengths. First, the effects of PMS were evaluated using both subjective clinical outcomes and objective anorectal physiological assessments, providing a more comprehensive evaluation of treatment response. In addition to assessing constipation symptoms and quality of life, high-resolution anorectal manometry was used to evaluate anorectal motor and sensory function, allowing us to investigate whether the clinical improvements were accompanied by changes in anorectal physiology. Secondly, a standardized PMS protocol was applied throughout the intervention, including consistent stimulation parameters and treatment procedures, which may improve the reproducibility of the findings and provide a reference for future studies investigating PMS in patients with FDD.

Several limitations should be acknowledged in the present study. First, the retrospective design and lack of a control group limited the ability to establish causal relationships between magnetic stimulation and clinical improvements. Although significant improvements were observed after treatment, the possibility of spontaneous symptom fluctuation or other uncontrolled confounding factors cannot be completely excluded. Future prospective controlled studies are needed to further validate the therapeutic efficacy of magnetic stimulation in patients with FDD. Second, the small sample size may have limited the statistical power to detect potential changes in certain anorectal physiological parameters, particularly RAIR-related outcomes. Larger-scale studies are warranted to confirm these findings and identify potential predictors of treatment response. Third, although the stimulation protocol used in the present study was based on previous clinical evidence, the optimal stimulation parameters, including frequency, intensity, and treatment duration, remain to be established. Further studies comparing different stimulation protocols are required to optimize therapeutic strategies. Finally, this was a single-center study with a relatively short follow-up period, which may limit the generalizability and assessment of long-term efficacy. Multicenter prospective studies with larger cohorts and extended follow-up are needed to further evaluate the clinical value and durability of magnetic stimulation for FDD.

## 5. Conclusions

In this uncontrolled retrospective study, patients with FDD showed significant improvements in constipation symptoms, quality of life, anorectal motility, and rectal sensory function following PMS treatment. These findings suggest that PMS may improve defecatory dysfunction by modulating anorectal sensorimotor function. Given its non-invasive nature and favorable safety profile, PMS may represent a promising therapeutic approach for FDD. Further prospective, controlled, and large-scale studies are warranted to confirm its clinical efficacy and optimize treatment protocols.

## Figures and Tables

**Table 1:** Patient characteristics.

Characteristics		
Gender	Male	12
	Female	9
Age/years		57.71 ± 15.46
Disease duration/years		5.86 ± 3.75

**Table 2:** Comparison of pre- and post-treatment CSS.

	Pre-Treatment	Post-Treatment	*p*-Value
Total CSS	15.29 ± 4.02	13.48 ± 4.86	0.045 *
Defecation straining	3.52 ± 0.60	2.81 ± 1.03	0.004 **
Feeling incomplete evacuation	2.91 ± 0.89	2.67 ± 0.97	0.234
Abdominal pain	0.81 ± 0.75	0.67 ± 0.86	0.452
Score of time in lavatory per attempt	2.67 ± 0.86	2.19 ± 0.98	0.038 *
Type of assistance	1.19 ± 0.60	0.95 ± 0.59	0.056
Unsuccessful attempts for evacuation per 24 h	2.05 ± 0.97	1.95 ± 0.97	0.493

Note: *—*p* < 0.05. **—*p* < 0.01.

**Table 3:** Comparison of anorectal motility parameter between pre- and post-treatment.

	Pre-Treatment	Post-Treatment	*p*-Value
ARP/mmHg	79.12 ± 19.55	68.69 ± 19.12	0.019 *
MASP/mmHg	147.10 ± 53.01	130.93 ± 45.24	0.071
RDP/mmHg	47.57 ± 20.59	44.28 ± 14.11	0.311
ARPd/mmHg	103.72 ± 48.89	73.87 ± 22.1	0.001 **
ARG/mmHg	−56.15 ± 48.11	−29.59 ± 22.54	0.006 **
ARR/%	−36.6 ± 65.7	−12.6 ± 39.9	0.046 *

Note: *—*p* < 0.05. **—*p* < 0.01. ARP: anal resting pressure, MASP: maximum anal squeeze pressure, RDP: rectal defecation pressure, ARPd: anal residual pressure, ARG: anorectal pressure gradient (difference between rectal pressure and anal pressure), ARR: anal relaxation rate during simulated defecation.

**Table 4:** Comparison of anorectal sensory threshold between pre- and post-treatment.

	Pre-Treatment	Post-Treatment	*p*-Value
first sensation volume/mL	32.57 ± 13.31	29.52 ± 10.98	0.234
desire-to-defecate volume/mL	58.57 ± 19.19	57.52 ± 18.33	0.744
urge-to-defecate volume/mL	92.1 ± 34.73	86.38 ± 24.45	0.778
maximum tolerable volume/mL	169.62 ± 47.29	146.9 ± 44.67	0.021 *
RAIR residual pressure/mmHg	40.29 ± 22.08	32.33 ± 16.48	0.061
RAIR relaxation rate/%	0.49 ± 0.17	0.54 ± 0.17	0.145
RAIR relaxation duration/s	16.71 ± 4.76	16.67 ± 3.4	0.968

Note: *—*p* < 0.05. RAIR: Rectal anal inhibitory reflex.

## Data Availability

The datasets generated for this study are available from the corresponding author.

## Abbreviations

The following abbreviations are used in this manuscript:

FDD	Functional defecation disorders
PMS	Peripheral magnetic stimulation
CSS	Constipation scoring system
PAC-QOL	Patient assessment of constipation quality of life
HAMA	Hamilton anxiety scale
HAMD	Hamilton depression scale
HRAM	High-Resolution Anorectal Manometry
ARP	Anal resting pressure
MASP	Maximum anal squeeze pressure
RDP	Rectal defecation pressure
ARPd	Anal residual pressure
ARG	Anorectal pressure gradient
ARR	Anal relaxation rate during simulated defecation
RAIR	Rectoanal inhibitory reflex
